# Molecular representations in AI-driven drug discovery: a review and practical guide

**DOI:** 10.1186/s13321-020-00460-5

**Published:** 2020-09-17

**Authors:** Laurianne David, Amol Thakkar, Rocío Mercado, Ola Engkvist

**Affiliations:** 1 Hit Discovery, Discovery Sciences, BioPharmaceuticals R&D, Astrazeneca Gothenburg, Sweden; 2 Department of Chemistry and Biochemistry, University of Bern, Bern, Switzerland

**Keywords:** Molecular representation, Cheminformatics, Drug discovery, Small molecules, Macromolecules, Linear notation, Molecular graphs, Reaction prediction, Artificial intelligence

## Abstract

The technological advances of the past century, marked by the computer revolution and the advent of high-throughput screening technologies in drug discovery, opened the path to the computational analysis and visualization of bioactive molecules. For this purpose, it became necessary to represent molecules in a syntax that would be readable by computers and understandable by scientists of various fields. A large number of chemical representations have been developed over the years, their numerosity being due to the fast development of computers and the complexity of producing a representation that encompasses all structural and chemical characteristics. We present here some of the most popular electronic molecular and macromolecular representations used in drug discovery, many of which are based on graph representations. Furthermore, we describe applications of these representations in AI-driven drug discovery. Our aim is to provide a brief guide on structural representations that are essential to the practice of AI in drug discovery. This review serves as a guide for researchers who have little experience with the handling of chemical representations and plan to work on applications at the interface of these fields.
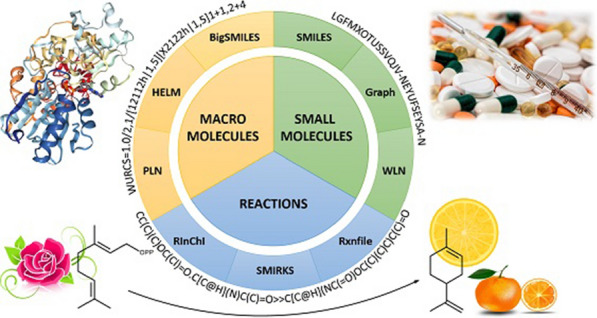

## Introduction

The representation of molecules has been of interest to scientists since the nineteenth century [1, 2]. Traditionally, molecules are represented as structure diagrams with bonds and atoms, and this is likely the representation most people think of when they think of molecules. However, other representations are required for the computational processing of chemical structures in cheminformatics. Here, we define a chemical representation as any encoding of a chemical compound; linear representations are referred to as notations.

Over the years, scientists have developed many notations depicting various properties of a compound. A classic notation is the empirical formula, a non-standard form of Hill notation. Seemingly simple at first glance, the empirical formula of alanine, C_3_H_7_NO_2_, exemplifies the complexity of building a notation. Indeed, while information about the atoms is available, it is not possible to know how the atoms are linked from the molecular formula which, moreover, does not encode information related to the molecular geometry. As such, the molecular formula above can be associated to alanine as well as to sarcosine and lactamide. Variants of empirical formulas emphasizing any functional groups also exist but are loosely defined. In these group-centric representations, elements are grouped in the formula as they would be in the molecule so as to highlight any functional groups present e.g. CH_3_CH(NH_2_)COOH to represent alanine.

The advent of computers led to the development of a wide variety of machine-readable chemical representations. Computers allowed for the rapid digital storage and querying of compounds and their structures, swift modifications of digital information, and greater physical storage efficiency. Algorithms were implemented to visualize compounds as 2D depictions [3, 4] and the computational visualization of compounds in 3D space was popularized with the development of specialized programs [5–7].

Many precursors to computer-readable notations were introduced between 1947 and 1964 and were dedicated to small organic molecules [2, 8]. At the time, memory efficiency was an important factor impacting the development of chemical notations. Popular representations used nowadays, however, were largely developed in and since the 70′s to represent small molecules [9–11], macromolecules [12–17] and chemical reactions [18–21].

In this review, we focus on chemical representations in cheminformatics and drug discovery. We first introduce the concept of a molecular graph, which is the most common machine-readable representation, and we give a brief overview of the main notations which paved the way for the current cheminformatics notations. We then focus on the representations that are used nowadays in the field of applying artificial intelligence (AI) to cheminformatics and drug discovery. Finally, we provide examples of AI-related applications using the chemical representations discussed in this review. This review is intended to provide an overview of basic cheminformatics knowledge to practising cheminformaticians, students in chemistry, cheminformatics, bioinformatics, and computer science, and anyone interested to learn more about molecular representations in drug discovery. While the coverage of representations introduced herein is not intended to be exhaustive, we emphasise that the representation used to solve a problem is always dependent on the task. Thus, the coverage is limited to areas where there is active research in applying machine learning (ML) and AI to cheminformatics and drug discovery. For readers interested in further reading on these topics we recommend references [22–29], in addition to the references cited in each section.

## Graph representations for small molecules

### Introduction to the molecular graph representation

In order to understand the chemical representations presented in this review, it is important that the reader first has a solid understanding of molecular graphs, as most molecular representations discussed in this work are built on the molecular graph representation. However, there is a distinction between the notations and file formats built using molecular graphs (e.g. SMILES strings, Molfiles), and the abstract mathematical structure/data structure of a graph itself. The latter is introduced here.

The idea behind the molecular graph representation lies in mapping the atoms and bonds that make up a molecule into sets of *nodes* and *edges*. Intuitively, one could imagine treating the atoms in a molecule as *nodes* and the bonds as *edges*, although there is no reason one could not consider other mappings. In typical graph representations, the nodes are represented using circles or spheres, and the edges using lines. In the case of molecular graphs, the nodes are instead often represented using letters indicating the atom type (as on the periodic table), or simply using points where the bonds meet (for carbon atoms).

A molecular graph representation is formally a 2D object that can be used to represent 3D information (e.g. atomic coordinates, bond angles, chirality). However, any spatial relationships between the nodes must be encoded as node and/or edge attributes, as nodes in a graph (the mathematical object) do not formally have spatial positions, only pairwise relationships. There are of course limitations to this representation, which are discussed in a later section. The 2D and 3D representations of graphs can easily be visualized by many software packages, including ChemDraw [30], Mercury [31], Avogadro [32], VESTA [33], PyMOL [34], and VMD [35] (the latter 5 are suitable for small- and macro-molecules, and either free or open-source).

### Mathematical definition of a graph

A graph^1^ is formally defined as a tuple *G* = (*V*, *E*) of a set of nodes *V* and a set of edges *E*, where each edge *e* ∊ *E* connects pairs of nodes in *V*. In a molecular graph, *V* is intuitively the set of all atoms in a molecule, and *E* is the set of all bonds linking the atoms, although this does not have to be the case. Molecular graphs are generally undirected, meaning that the pairs in *E* are unordered. [36].

To map a graph from an abstract mathematical concept to a concrete representation that can be handled on a computer, one needs to map the sets of nodes and edges to linear data structures; a common way to do this is using data structures such as matrices or arrays. Linear data structures are necessary in order to specify the connectivity of the nodes. To do so, an artificial node-ordering must first be calculated for encoding a molecule using arrays, even though *V* and *E* are formally sets and the order of elements in sets is irrelevant. The information to be mapped can include (1) how the atoms in the molecule are connected, (2) the identity of the atoms, and (3) the identity of the bonds.

**Fig. 1:**
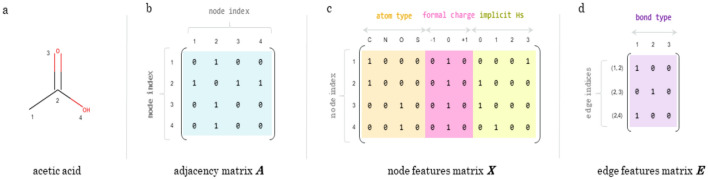
Example graph representation for acetic acid. **a** Graph representation of acetic acid with nodes numbered from one to four. **b** Example adjacency matrix, ***A***, for an acetic acid graph with the corresponding node ordering on the left. **c** Example node features matrix, ***X***, which one-hot encodes a few selected properties. **d** Example edge features matrix, ***E***, where each edge feature vector is a one-hot encoding of single, double, or triple bonds. “Implicit Hs” stands for the number of implicit hydrogens on a given node

How the atoms are connected is commonly represented in the form of an *adjacency matrix* ***A***; given that *a*_*ij*_ is an element of ***A***, *a*_*ij*_ = 1 means that there exists a bond between nodes *v*_*i*_ and *v*_*j*_ in molecular graph *G*, whereas *a*_*ij*_ = 0 means that there exists no bond between them (Fig. 1b). The adjacency matrix is also sometimes referred to as the *connectivity matrix*. Note that the adjacency matrix does not necessarily specify what type of bond is connecting each pair of nodes.

The identity of the atoms can be represented in the form of a *node features matrix* ***X*** (Fig. 1c). Each row of ***X*** corresponds to a node *v*_*i*_ (i.e. an atom in the molecule) in *G*; this row is also referred to as the *node feature vector* ***x***_***i***_ for that atom. The length of ***x***_***i***_ corresponds to the number of atom features one has chosen to encode (e.g. a one-hot^2^ encoding of atom type and formal charge).

The identity of the bonds can be represented in the form of an *edge features matrix* ***E*** (Fig. 1d). Each row of ***E*** corresponds to an edge *e*_*ij*_ = (*v*_*i*_, *v*_*j*_) in *G*, and is referred to as the *edge feature vector* ***e***_***ij***_ for that edge. The length of ***e***_***ij***_ corresponds to the number of edge features one has chosen to encode (e.g. a one-hot encoding of possible bond types {single, double, triple, aromatic}).

Although common in AI applications, we would like to point out that it is not necessary to one-hot encode the various node and edge features. For example, the node features matrix shown in Fig. 1c could instead have only 3 columns using integers to represent the same three properties (atom type, formal charge, and number of implicit Hs).

### Graph traversal algorithms

**Fig. 2:**
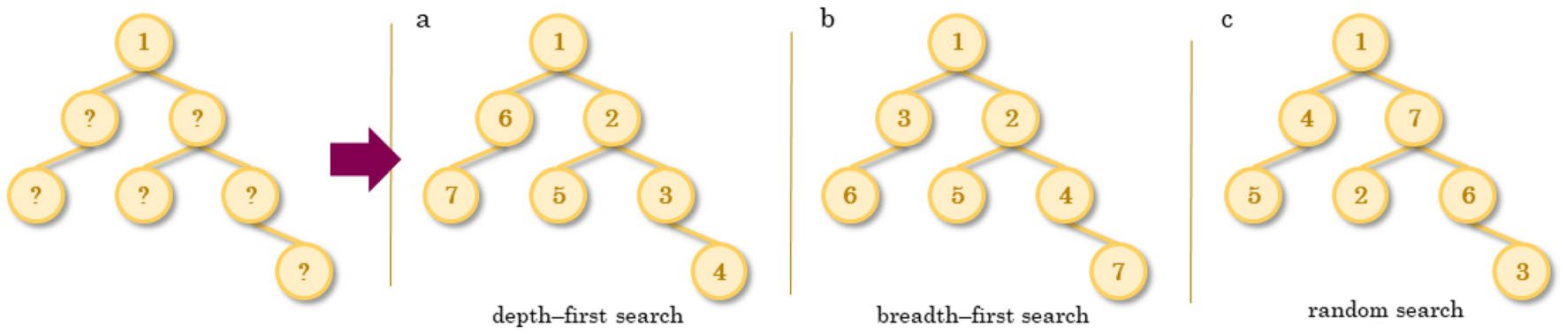
Graph traversal algorithms. Three widespread graph traversal algorithms are illustrated above for an example branched graph. The numbers correspond to the order in which the nodes are explored, starting at node 1. **a** A depth-first search first explores each “branch” of a graph to the fullest extent, then goes back and explores branches at the last branched node, until all branches have been explored. **b** A breadth-first search first explores all nearest neighbours of a node, and then the nearest neighbours of the nearest neighbours, and so on, until the whole graph has been explored. **c** A random search explores nodes in the graph in an arbitrary order, regardless of how they are connected

Although, formally, graphs are non-linear data structures made up of sets of nodes and edges, in practice, matrix representations of graphs are node order dependent. The node order used in a matrix representation is determined by a graph traversal algorithm (Fig. 2). Depending on the application, it can be desirable to consistently generate the exact same representation for the same molecule. Reliably generating the same representation for a molecule is dependent on getting the same node order every time. To this end, one can use methods such as a depth-first or breadth-first search to generate graph matrix representations. The graph traversal algorithm needs to include a consistent way to break ties when a node branches off and must therefore consistently select the same branch traversal order. In fact, the way in which different software packages break ties in traversing a graph is often what sets them apart. However, if consistency is not important (and, indeed, for some deep learning applications, one might want noisier data), a random search can be used.

### There are many ways to represent a graph

The matrix representations discussed above are not the only way to represent graphs, as there are multiple ways to represent the same information. For example, as has been already mentioned, depending on what graph traversal algorithm is used, the order of the rows in the adjacency matrix (or atom/bond block) will be different.

Furthermore, when working with molecular graphs, there is not one correct way to represent any molecule, and the representation chosen must be appropriate for the task.

### Advantages of molecular graph representations

Graphs are formally 2D data structures with no spatial relationships between elements; nonetheless, 3D information (and information that is the “result” of a 3D structure e.g. stereochemistry) can be encoded into a graph representation. One natural place to put this data is in the node features matrix, ***X***, for node information (such as if a chiral node is R or S), or in the edge features matrix, ***E***, for edge information (such as the length of a bond).

The fact that one can naturally encode 3D information in a graph representation gives graphs many advantages over various linear notations, although some linear notations (such as SYBYL Line Notation) can also encode atomic 3D information. Additionally, the fact that all molecular subgraphs (i.e. subsets of *G*) are *interpretable* can confer a particular advantage to graph notations over certain string notations, where, for example, substrings of a SMILES string (which we describe in the next section), do not necessarily correspond to a valid graph. In other words, all subgraphs are interpretable whereas all substrings are not. Nonetheless, there are also disadvantages to working directly with the molecular graph representation for many applications.

### Limitations of molecular graph representations

#### Breakdown of graph model

There are many types of molecules which cannot be described by the graph model. This is any structure containing any form of delocalized bonds, such as coordination compounds, as well as any molecule containing any of the following: polycentric bonds, ionic bonds, or metal–metal bonds. For example, organometallic compounds such as metallocenes or metal carbonyl complexes are difficult to describe using molecular graphs because their bonding scheme cannot be explained by valence bond theory. In other words, it would be difficult to describe the bonds using only pairwise relationships between atoms.

Solutions to the handling of multi-valent bonds have been introduced via the use of *hypergraphs*; in a hypergraph, edges are sets of at least two atoms (*hyperedges*) instead of tuples of atoms [37]. However, the use of hypergraphs is not further discussed here as they are not currently widespread in the field.

For molecules where the arrangement of atoms is constantly changing in 3D space, the graph representation might not be meaningful, especially if pairwise bonds are breaking and forming or if the structure is frequently rearranging. That is, for applications where one is limited to using a single static representation for a molecule that is in fact rearranging on the timescale of the problem (e.g. tautomers), then a single molecular graph representation would not be appropriate and could even be detrimental to solving the problem.

#### Challenges of working directly with the graph representation

Another difficulty of working directly with graph representations is that they are not *compact* (both memory-wise and literally). To represent a molecular graph one would need, for example: an image, a tuple of matrices, lists, or tables; all these representations are generally more difficult to search through than a more compact linear representation (compare this to a string encoding a structure ID). They also become more and more cumbersome the bigger the graphs get, and their memory requirement would increase with the square of the number of nodes, at least.

This is not a problem with linear notations, which build upon the graph framework to create more compact and memory-efficient representations for molecules [38]. Linear notations have the advantage that they can be, for example, entries in a table, as well as easily searchable (for identity search, not substructure search), when a matrix representation is not convenient.

### Connection tables and the MDL file formats

Below we discuss two formats closely related to the molecular graph representation: connection tables and the MDL (now BIOVIA) file format.

#### Connection tables

Whilst graphs underlie the representation of molecules, the matrices by which they are described are not a compact representation, and scale as the square of the number of atoms. The connection table (Ctab) [39] is composed of six parts: (1) *Counts line*, (2) *Atom block*, (3) *Bond block*, (4) *Atom list block*, (5) *Stext block*, and (6) *Properties block*. Readers are referred to the referenced material for a detailed description of each component. The counts line is always the first line, and as such gives an overview of the structure by specifying the number of atoms, bonds, and atom lists, as well as the presence or absence of chirality. The version (V2000 or V3000) is also specified on this line. The *atom* block describes the identities of the atoms as a list with arbitrary index values, as well as the atomic symbols, mass differences, charge, stereochemistry, and associated hydrogens. Note that it is often practical to treat any hydrogens in a molecule as *implicit*—that is, not storing hydrogens as atom objects and instead implicitly defining the hydrogen count using a valence model. Treating hydrogens as properties of the heavy atoms rather than as explicit nodes significantly reduces the size of the atom and bond block, making the format more compact. These can be recalculated based on valence rules if required; in such cases, valency information must be given explicitly in the atom block. The *bond block* describes the connectivity of the atoms as well as the identity of the bonds connecting them. The atoms may be fully or partially connected by bonds, thereby supporting the description of fragments and unconnected atoms. The *bond block* is composed of the atom indices and bond types; the bond order is also provided as an additional column. There is no requirement for the bond block of the connection table to be ordered in a particular way. The two blocks are combined to form the core of the Ctab. Similarly to a matrix representation, the Ctab is extensible, meaning that lists describing supported properties may be added to the properties block. Notably, any entries associated with charges, radicals, or isotopes supersede those in the atom block, if present. As a result of backwards compatibility to previous versions and the prevalence programs utilising them, connection tables have become one of the standard formats for handling chemical structural information and underlie the widely used Molfile formats. It should be noted that connection tables are in themselves not a file format but are the core building block around which CT files are built.

#### The Molfile format

**Fig. 3:**
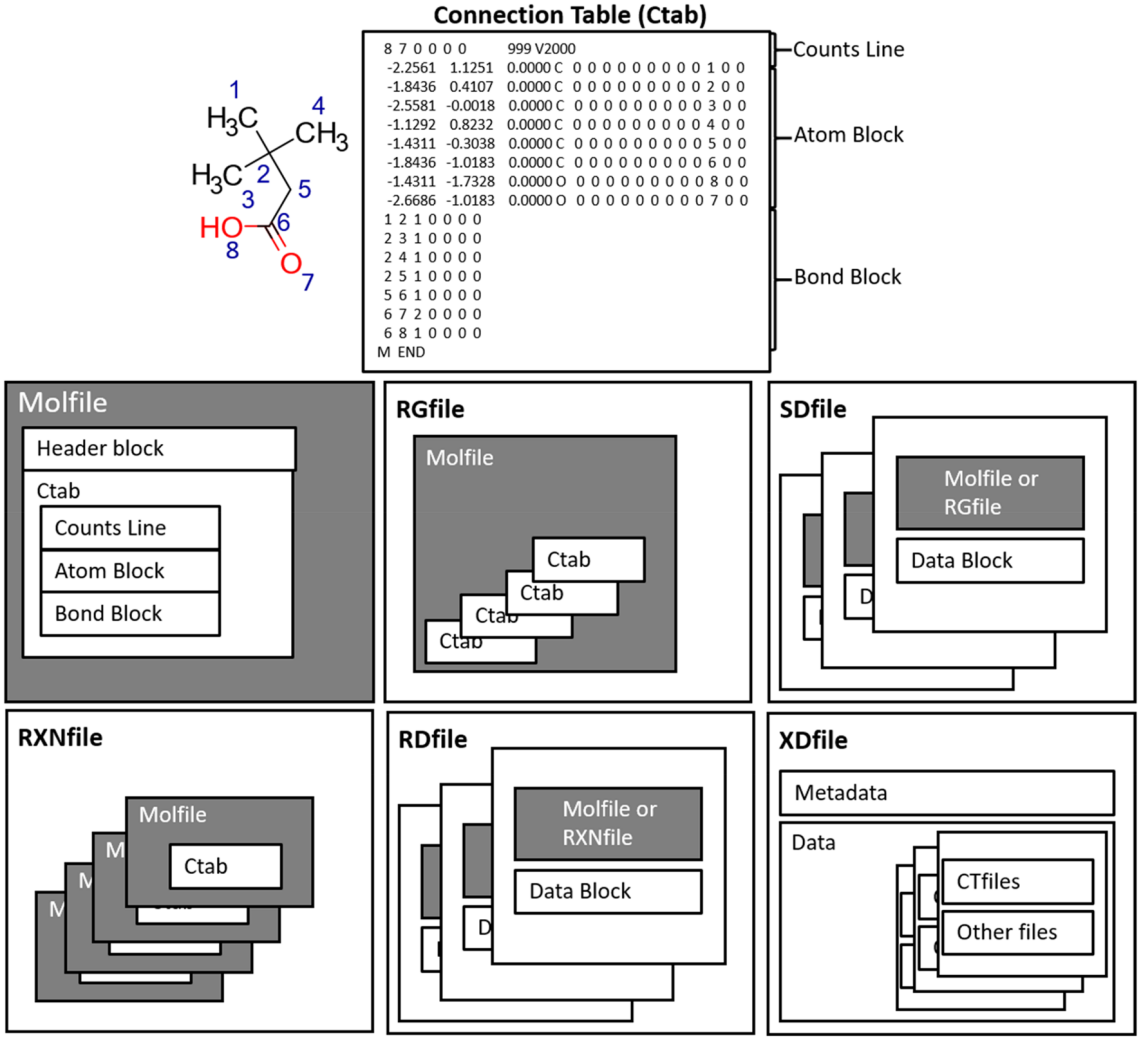
The MDL family of file formats are collectively known as CTfiles (chemical table files) as they are built upon connection tables (Ctab), shown at the top of the figure. The connection table is split into an atom and bond block, describing the atoms and their corresponding connectivity. The Ctab is built upon to form the Molfile for the description of single molecules, RGfile for handling queries, SDfile for structure and associated data, RXNfile for the description of single reactions, RDfile for either a series of molecules/reactions and their associated data, and the XDfile for the transfer of structure or reaction data based on the XML format

The Molfile format family developed by MDL are collectively known as CTfiles (chemical table files) as they use connection tables to describe molecular structures. In addition, CTfiles are highly extensible and as such have formed a series of file formats that have been widely used for chemical information transfer. The series is shown in Fig. 3, which shows how the connection table is wrapped within the Molfile format, which can be subsequently wrapped into a structure/data (SD) file, containing both structural information and additional property data for any number of molecules.

Similarly, the RXNfile contains the description of single reactions and the RDfile enables storage of reactions or molecules as well as their associated data. RGfiles on the other hand have been designed for handling queries and the XDfile is an XML based format for the transfer of structures or reactions along with their associated metadata. Further details about each of the files and their structure can be found in the MDL documentation and various textbooks introducing the field of cheminformatics [40].

## Linear notations for small molecules

Matrix representations require a large amount of disk space and are not well adapted for basic cheminformatic analysis (i.e. generation of list of compounds, online query of compounds). As a result, molecules nowadays are often represented as strings of characters encoding the Ctab and that can be interpreted by systematic sets of rules. For example, using implicit hydrogen, representing d-alanine using a Molfile takes 612 bytes, while using linear notations such as SMILES or InChI, which are described in this section, takes 15 and 59 bytes, respectively. As mentioned above, linear notations have the advantage of being compact and easy to manipulate (e.g. to use as command-line option or copy in an Excel spreadsheet). The main linear notations introduced in this section are exemplified in Table 1.

**Table 1:** Examples of chemical representations of alanine

Notation system	Handles stereochemistry?	Representation
2D depiction	Yes	
Generic name	Yes	d-Alanine
IUPAC name	Yes	(2R)-2-aminopropanoic acid (English) acide (2R)-2-aminopropanoïque (French) (2R)-2-aминoпpoпaнoвaя (Russian)
WLN^a^	No	QVYZ [46]
SMILES	No Yes	
InChI	No Yes	InChI = 1S/C3H7NO2/c1-2(4)3(5)6/h2H,4H2,1H3,(H,5,6) InChI = 1S/C3H7NO2/c1-2(4)3(5)6/h2H,4H2,1H3,(H,5,6)/t2-/m1/s1
InChI Key	Yes	QNAYBMKLOCPYGJ-UWTATZPHSA-N
HELM	Yes	PEPTIDE1{[dA]}$$$$
Three-letter symbol	Yes	D-Ala
Protein Line Notation (PLN)	Yes	H-{d}A-OH

Colour coding on the alanine 2D depiction matches the one of SMILES representations

^a^Q = OH, V = carbonyl, Y = branching, Z = NH_2_, < digits > = unbranched alkyl chain(s)

### The IUPAC quest for a universal notation

Over time, the way scientists name molecules has varied following the capabilities and needs of the scientific community. In the ages of alchemy, compounds and elements were named based on their properties; for example, *aqua fortis* and *sweet oil of vitriol* referred to nitric acid and diethyl ether, respectively. In the nineteenth century, the need for a systematic nomenclature of organic chemistry grew stronger, and a terminology was developed by the International Union of Pure and Applied Chemistry (IUPAC) [41]. This terminology is described at length in the IUPAC Color Books [42] and is universally used in the literature, patents, and government legislation. Nonetheless, this nomenclature is not ideal for cheminformatics applications and, in 1949, the IUPAC requested an international standard for electronic chemical notations requiring 11 desirable properties or “desiderata” [28]: simplicity of use, ease of printing and typewriting, conciseness, recognizability, ability to generate a unique chemical nomenclature, compatibility with the accepted practices of inorganic chemical nomenclature, uniqueness, generation of an unambiguous and useful enumeration pattern, ease of manipulation by machine methods, exhibition of associations, and ability to deal with partial indeterminates.

According to the IUPAC formalism set in 1964 [28], notations can be classified as being unique (i.e. one notation for a given compound), non-unique (i.e. more than one notation for a given compound), ambiguous (i.e. the notation will regenerate more than one compound), or/and unambiguous (i.e. the notation will regenerate only the original compound). This formalism is used to describe notations in this section.

Although seven notations^3^ were proposed to IUPAC as potential standards, only two retained the interest of the committee: the Dyson cyphering [43] and the Wiswesser Line Notation (WLN) [44]. Descriptions and related references for the remaining five notations can be found in two notable publications [2, 45] which detail many chemical notations introduced until 1984, and in a report by Alan Gelberg [8]. After many revisions, Dyson’s notation, originally developed in 1947, was adopted in 1961 as an international notation by IUPAC. The Dyson cyphering was not very popular among the scientific community as it could not be handled on standard typewriters or ordinary punched-card machines and contained many arbitrary rules. The most used notation by the community was WLN, which was created in 1949 and did not present the drawbacks of the Dyson cyphering. For a detailed comparison between WLN and Dyson cyphering, we refer the readers to the Survey of Chemical Notations [28]. We do not provide further details on WLN and IUPAC-Dyson as the notations have fallen into desuetude; however, we feel that the competition between both notations illustrate the technological and technical aspects that were under consideration for the selection of a universal notation.

### The advent of contemporary notations

#### Simplified Molecular Input Line Entry System

WLN requires an extensive knowledge and understanding of the notation’s rule. A more intuitive notation, the *Simplified Molecular Input Line Entry System* (SMILES), was developed in 1988 by Weininger et al. [9] and has been the most popular line notation ever since. SMILES notation system was then incorporated into the Daylight Chemical Information Systems [48] toolkit; the company is still currently maintaining it. The SMILES representation, non-unique and unambiguous, is obtained by assigning a number to each atom in the molecule and then traversing the molecular graph using that order; in the case of RDKit [49], the graph traversal algorithm used is depth-first search.

**Fig. 4:**
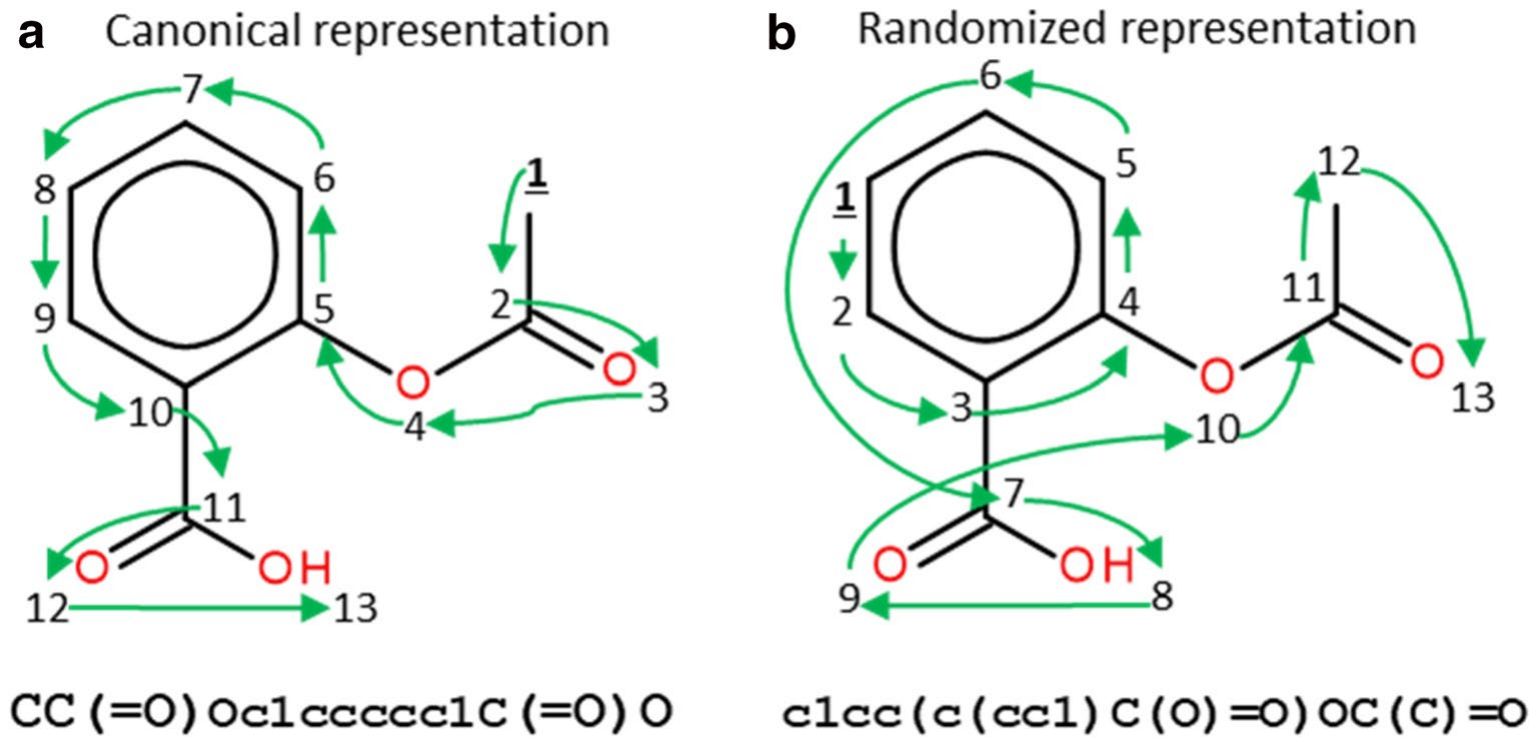
Canonical (**a**) and randomized (**b**) SMILES representations of aspirin. Randomized SMILES correspond to the various representations of a molecule obtained by randomly selecting the starting node in the graph traversal algorithm, thus changing the order of the nodes traversed in the molecular graph (still using depth-first search). Numbers represent the order of graph traversal, where 1 is the initial node (user defined). Considering **a** as being the canonical representation of aspirin, **b** shows a different ordering of the atoms of the molecule. The final SMILES is one possible SMILES among all the randomized SMILES which can be generated. Green arrows indicate how the molecular graph is traversed. Both SMILES strings shown represent the same molecule but, as the atom numberings are different, the generated SMILES strings are, too. The original figure can be found in [47]

There can be multiple atom numberings for a given molecule, leading to different SMILES. SMILES can thus be enumerated for data augmentation [50]. The ensemble of SMILES representing one molecule can be referred to as enumerated or randomized SMILES and are obtained by, for each molecule, randomly selecting an initial node for graph traversal while keeping the same graph traversal algorithm, thus leading to different atom orderings [51]. For clarity, we emphasize that randomized SMILES do not use a random search to generate representations, they rely on a depth-first search. To avoid conflicting SMILES representations for the same molecule, a unique SMILES can be designated, and several canonicalization methods exist to this end [38, 52, 53]. A schematic illustrating the difference between two SMILES variants is shown in Fig. 4.

Initially, SMILES did not encode for stereochemistry. A specification, referred to as isomeric SMILES, was introduced later on and is now the default SMILES in many software. SMILES can thus encode isomeric specifications, configurations around double bonds (Z or E), and configurations around tetrahedral centres as well as many other types of chiral centres which are rarely supported (e.g. allene-like, octahedral). Nonetheless, a problematic set of structures to describe using SMILES notation is those which cannot be easily described using molecular graphs (see “Limitations of molecular graph representations” section), such as organometallic compounds and ionic salts.

Generally, if the total sum of bond orders is not equal to one of the standard valences for a given atom in a molecule, this is addressed in the corresponding SMILES notation using square brackets. When the molecules involved in bonding are also aromatic, lowercase tokens may be used, though this becomes problematic with some cheminformatics software which do not allow “extra” bonds for aromatic atoms [54]. ChemAxon Extended SMILES (CXSMILES) can overcome some of these issues by storing special features [55]. These are stored after the SMILES string separated by a space or tab and can be ignored when parsing SMILES if necessary. In addition, several fields can be stored for any given SMILES string. One such feature is fragment grouping, which specifies which components are grouped together using a list of fragment indexes; this aids in the grouping of ions and salts. Additional specifications of ligand order and coordinate bonds aid in the description of organometallic compounds and are supported by CXSMILES. The atom-to-atom coordinate bonds are represented by single bonds in the SMILES but corrected by the additional information CXSMILES provides in the extension.

The OpenSMILES [56] specification was developed in 2007 to provide a SMILES standard form and to clarify some interpretations of corner cases present in the Daylight’s SMILES system. A major issue with Daylight’s SMILES is that its canonicalization algorithm is proprietary, and as such implementations vary between companies and research teams. A novel open source method to generate canonical SMILES was developed in 2012 [38]. Such SMILES are generated using the canonical label provided by the InChI representation [10], which is described later in this section. Using such “universal” SMILES seeks to facilitate the comparison between chemical models used by different toolkits.

#### SMILES Arbitrary Target Specification (SMARTS)

There exists an extension of SMILES developed for substructure searching, named *SMILES Arbitrary Target Specification* (SMARTS) [57]. In SMILES there exist two types of symbols to signify atoms and bonds which describe the underlying connectivity of a given molecular graph. However, in SMARTS, the available symbols allow for a more general specification of the molecular graph. This can be likened to the use of regular expressions in computer science. Classical SMARTS can describe an ensemble of molecules that differ at one atom or bond position. It is also possible to include logical operators such as “OR” and “NOT”. Contrary to SMILES, SMARTS can specify different isotopes or bonds types (aromatic or aliphatic). Detailed information about an atom environment can be given using Recursive SMARTS (e.g. ortho, meta, or para substitution patterns in arenes). All SMILES can be valid SMARTS, however the reverse is not true, and decoding a SMILES as a SMARTS will generally not yield the same decoded pattern.

#### International chemistry identifier

**Fig. 5:**
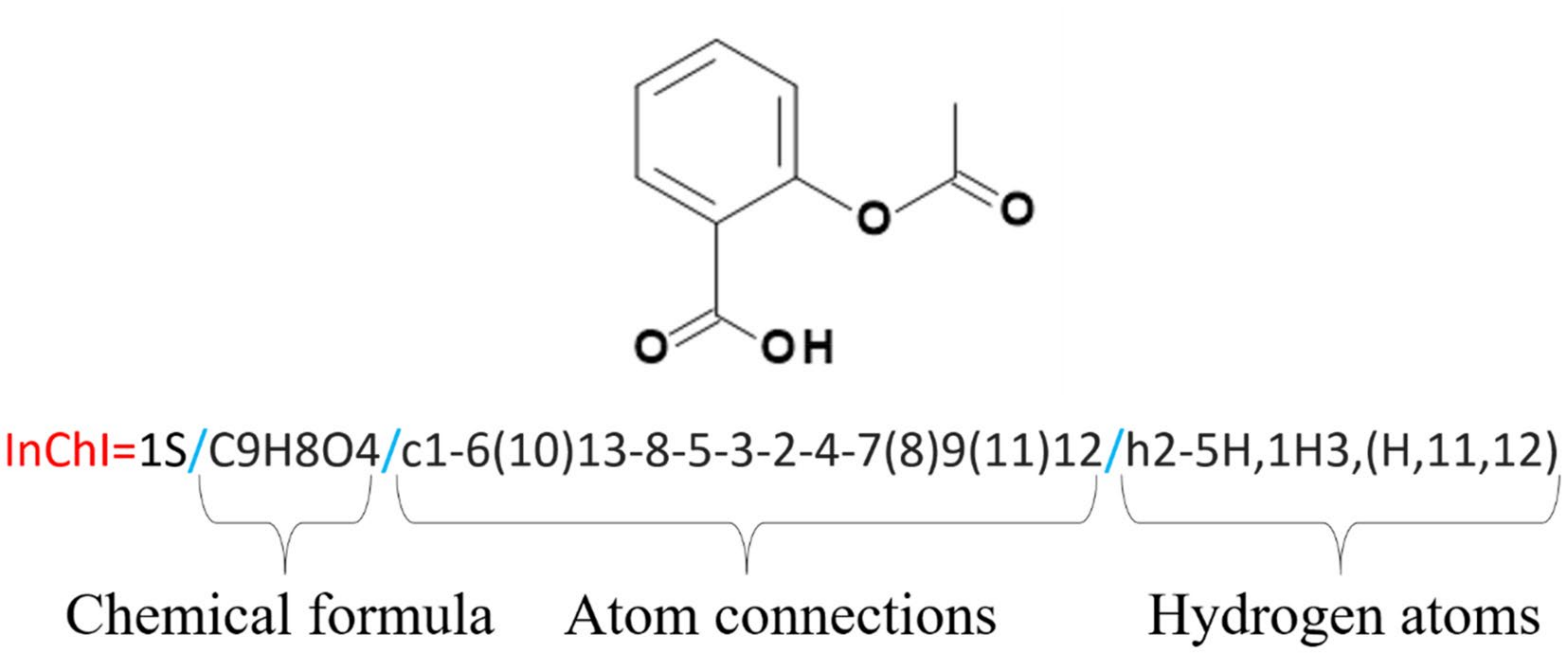
InChI notation of aspirin. Red letters are the standard beginning of the notation. The following 1 corresponds to the InChI version number, and S states that the notation is a standard InChI. Slashes (blue) are delimiters

The best example of open-source canonical notation is the InChI (International Chemistry Identifier) representation [10], which was introduced in 2006 by NIST, under the auspices of the IUPAC, as a standard and freely available formula representation. InChI are composed of multiple layers, such as the *Main*, *Charge*, *Stereochemical*, and *Isotopic* layers, to name a few, which are themselves constituted of sublayers. For example, the *Main* layer is composed of the *Chemical formula*, *Atom connections*, and *Hydrogen atoms* sublayers (Fig. 5).

A hashed version of the InChI, the InChIKey, is used for open-web searching and library searching [58]. The first block of an InChIKey represents the molecular skeleton, and the second block encodes for isomerism. InChIKeys are designed to be unique representations of their corresponding parent InChI representations. However, an InChIKey can sometimes map to more than one InChI, the situation being referred to as an InChIKey collision [59]. Unlike SMILES, InChIs are not guaranteed to be decodable back to the molecular graphs from which they originate, and SMILES have the advantage of being more human-readable. For a detailed overview of the applications of InChIs and the underlying algorithm, readers are referred to the works of Heller [10] and Warr [60].

### Using chemical descriptors to represent molecules

The representations presented above are atom-based, meaning it is possible to rebuild the molecule based on the representation. There exist, however, other types of notations which, rather than encoding the exact structure of a compound, instead encode the physicochemical, structural, topological, and/or electronic properties of a compound. These are referred to as *molecular descriptors* [11] among which two main classes are structural keys and the hashed fingerprints. Descriptors are unique and ambiguous notations widely used in cheminformatics, and their complete descriptions would require a review of their own. A non-exhaustive list can be found in association with the Dragon software [61], which can calculate 4885 descriptors.

#### Structural keys

Structural keys are bit strings, encoding for the absence (using a 0) and the presence (using a 1) of a specific chemical group. To provide a general understanding of the structural keys concept, we present here a few widely used keys.

*MACCS Keys* The first set of keys is referred to as MACCS (Molecular ACCess System) keys or MDL keyset [62, 63] and is frequently used for similarity searching. In MACCS keys, each bit indicates the presence or absence of a particular structural fragment. Many variants of the MACCS keys exist [64], with the most commonly used being 166 and 960-bits long. thus encoding for the presence or absence of 166 and 960 structural fragments. It should be noted that there are many software implementations of the 166-bit MACCS keys, thus one should be cautious as one substructure will not be assigned to the same bit from one software to the other.

*CATS* For application in scaffold hopping, a topological pharmacophore descriptor, Chemically Advanced Template Search (CATS) [65], was developed. It can encode for six potential pharmacophore points: H-bond donor/acceptor, positively/negatively charged, aromaticity, and lipophilicity.

#### Hashed fingerprints

Chemical fingerprints are vectors which contain indexed (ordered) elements encoding for physicochemical or structural properties. Hashed fingerprints differ from other descriptors by the fact that each feature is generated from the molecule itself, while in keys, patterns are pre-defined. Their lengths can be set prior to their generation and a hash function assigns molecular patterns to (non-unique) bits, hence the name. Topological or path-based fingerprints are represented by Daylight fingerprints, which usually consist of 512, 1024, or 2048 bits. The Daylight fingerprint encodes for every connectivity pathway within a molecule up to a given length. Circular fingerprints are representations of chemical structures by atom neighbourhoods and have been widely applied in Quantitative Structure–Activity Relationship (QSAR) analysis. A widely used class of circular fingerprints is ECFP (Extended Connectivity Fingerprints) [66], based on the Morgan algorithm. In ECFPs, heavy (i.e. non-hydrogen) atoms are encoded into multiple circular layers up to a given diameter.

Whether fingerprints can be called a chemical *notation* per say is debatable and comes down to a matter of opinion between experts. Regardless, chemical fingerprints are widely used in cheminformatics and drug discovery as they provide a quick and direct mapping from a graph to a vector representation that can be used as input to numerical models, such as QSAR models. It should be noted that fingerprints are flexible representations and can also encode physicochemical properties as integers (e.g. the hydrogen count) and floats (e.g. molecular weight).

## Representations for chemical reactions

### Harnessing reaction data for drug discovery

Chemical reactions represent the interconversion of one set of molecules into another related set, under a set of specified conditions. A vast body of reaction data has been amassed to date, with approximately 127 million reactions recorded from 1840 to the present day according to the Chemical Abstracts Service (CAS) [67].

In recent years, there has been a resurgence of interest in the development of models for the prediction of outcomes of chemical reactions, synthetic routes, and analysis of reaction networks, to name a few application areas. For a more comprehensive coverage of the representations of chemical reactions in databases and computer-aided synthesis design, we refer the reader to a review by Warr and the bibliography therein [68]. In addition, for a more comprehensive coverage of applications within autonomous discovery, we refer the reader to an extensive review by Jensen et al. [69].

Many of the representations described in the previous sections natively allow for, can be extended, or have analogous representations for describing chemical reactions. As the description of reactions is that of a set of molecules, limitations in each of the previously described representations are inherited in the description of chemical reactions. A reaction is often represented graphically with the *reactants* written to the left of a *reaction arrow*, and a set of resulting *products* written to the right of this arrow. The *conditions* under which the transformation occurs are written above or below the arrow, including information such as reagents, catalysts, solvents, temperature, and so forth. The graphical illustrations of reaction schemes often found in publications are, however, not easily machine-readable. Therefore, there exist a series of reaction data exchange formats that enable reactions to be represented in a machine-readable format. There is no inherent requirement for one format or another, as this is dependent on the application, toolkit, or software package used. Commonly used formats include the RXN and RD files described in an earlier section.

#### Reaction SMILES and SMIRKS

The SMILES format used for describing molecules has been extended to so-called Reaction SMILES by Daylight Chemical Information Systems. Each molecule in the reactants, agents, and products is represented by a SMILES string, and disconnected structures are separated by a period; this includes the individual molecules, ions and ligands, which are listed in an arbitrary manner. Reactants, agents, and products are separated by either the ‘>’ or ‘≫’ symbol (the latter used when agents are not given). Atom-mappings (i.e. mappings of atoms in the reactants to their equivalent atoms in the products) can be stored in Reaction SMILES as a non-negative integer following the character ‘:’ within an atom expression. Atom mappings do not apply to agents. Furthermore, the storage of additional textual information such as the reaction centre (i.e. the atom and bonds that change during a transformation) or reaction conditions is not supported. Nonetheless, formats such as the RXN and RD file formats, especially the latter, can store this additional metadata, as can other file formats or databases.

**Fig. 6:**
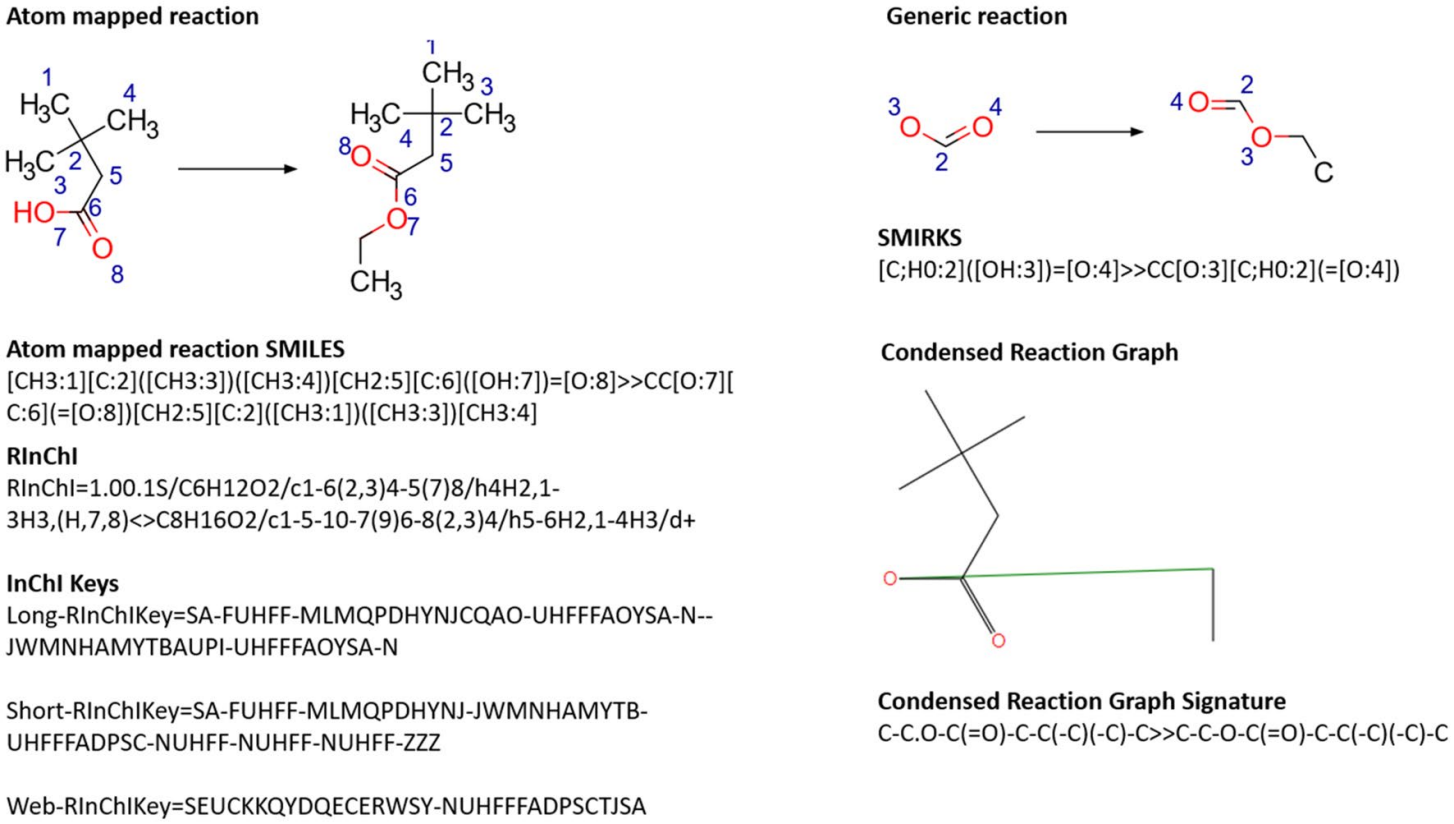
A selection of representations for a simple esterification reaction. The atom mapped reaction is shown in the top left as a structural diagram. The atom maps are consistent between reactant and product as shown. The atom maps in the SMIRKS do not correspond to the atom maps in the full reaction. Rather, they are used to keep track of the atoms within the SMIRKS. The condensed reaction graph and corresponding signature was generated using CGRtools [73]

SMIRKS belong to the same family as SMILES and SMARTS. Where SMARTS describe molecular patterns or substructures generically, SMIRKS patterns can be used to define generic reaction transformations. They can be used to describe the reaction centre, to enumerate virtual libraries, and to form the knowledge base for reaction and retrosynthetic prediction systems. If one considers that a reaction is a set of atoms and bonds that change during a reaction and the reactant or substrate upon which that change occurs, then SMIRKS must encode the same set of atoms and bonds that change during the reaction, and the site at which that change occurs in the substrate as specified by a SMARTS pattern. The SMARTS pattern is used to specify both the site at which the atom and bond changes occur, and to capture any indirect effects that may influence the reaction. The atomic expressions must be defined such that (a) for any part of a molecule that is to be considered in a generic transformation for which the bonding does not change, SMARTS are to be used, and (b) in cases where bonds change, SMILES are to be used. In this sense, SMIRKS is a hybrid approach between SMILES and SMARTS. There are some rules that must be followed in order to ensure that SMIRKS patterns can be applied. The two sides of the transformation, the reactant(s) and product(s), must contain the same number of mapped atoms, and they must correspond on either side of the reaction. Additionally, any explicit hydrogens must appear explicitly on either side of the reaction and have corresponding atom mapping numbers. SMIRKS are converted into a reaction graph for their subsequent use. The reaction SMILES and corresponding SMIRKS are shown in Fig. 6.

#### RInChI

An extension of the InChI, RInChI [18, 70], was developed between 2008 and 2018 and introduced a unique, order invariant identifier for reactions. It was developed in response to the growing size of reaction data to aid reproducibility, to consider more information than just the participating molecules, and to provide enough information such that practically identical reactions would be represented the same way. RInChI grammar, however, is relatively more complicated than that of Reaction SMILES.

RInChIs use InChIs to describe each molecule. Where InChIs cannot be generated for a molecule, the RInChI tracks the number of “structureless” entities that are present in each of the reactants, agents, and products. In addition to specifying each molecule and reaction role, the RInChI must include information about equilibrium, unbalanced, or multi-step reactions. The RInChI employs a layering system, whereby each layer can describe a different aspect of the chemical reaction. Solvents and catalysts may be accounted for in a similar manner as in Reaction SMILES; however, RInChIs additionally allow for the direction of the reaction to be described. This is particularly useful, as different labs may conduct the same reaction under slightly different conditions, potentially reaching different conclusions about the direction of the reaction. The RInChI generated in this case would be the same, except for the direction flag. This aids in the identification of reactions that are in practical terms identical.

A proposed further extension to RInChI, ProcAuxInfo, enables the storage of metadata relating to yields, temperature, concentration, and other reaction conditions [71]. RInChI offers an alternative to Reaction SMILES that enables the identification of duplicate reactions, as the order in which molecules are listed in Reaction SMILES is arbitrary. Hashing the RInChI to yield the RInChI key provides a powerful tool for efficiently indexing and searching reaction data [18, 71]. However, there is no SMARTS or SMIRKS equivalent for RInChI, limiting its use in substructure searching and in encoding generic chemical transformations. The RInChI and corresponding keys are shown in Fig. 6.

#### Condensed graph of reaction (CGR)

Varnek and co-workers have developed the CGR approach [19], whereby molecular structures are encoded in a matrix containing the occurrence of fragments of a given type. The CGR is a superposition of the reactant and product molecules, and additionally defines what atoms and bonds have changed as well as their properties. This builds on the description of organic reactions using imaginary transition states as described by Fujita [72]. In analogy to SMIRKS, the CGR can be used to describe a reaction transformation. An example CGR is shown in Fig. 6.

With the renewed interest in chemical reactions within cheminformatics in recent years, Varnek and co-workers have developed an open source toolkit enabling the wider use of CGR [73].

#### Bond electron matrices (BE-matrix)

To exemplify the representation of reactions as matrices, the bond-electron matrix developed by Dugundji and Ugi was previously employed for reaction classification and has also been used as an inspiration for the representation used in programs such as the Elaboration of Reactions for Organic Synthesis (EROS) [74], and the Workbench for the Organisation of Data for Chemical Applications (WODCA). The BE-matrix is an *N* by *N* matrix, where *N* is the number of atoms in a molecule, and the diagonal entries specify the number of free valence electrons. The off-diagonals specify the bond orders between atoms as found in the bond matrix. The reaction is represented by an “R-matrix” which corresponds to bond changes or changes of non-bonded valence electrons. Positive values indicate bond formation, whereas negative values indicate bond breakage. Adding the “R-matrix” to the BE-matrix of a reactant gives the BE-matrix of a product. The “R-matrix” is therefore an alternate method for representation of the reaction centre [20]. The BE-matrix illustrates the concept of adding additional information into the matrix representation.

#### Hierarchical organization of reactions through attribute and education (HORACE)

HORACE [21] employs a machine learning algorithm for the classification of chemical reactions and is mentioned here because of the hierarchical description of chemical reactions that it uses. It was developed to describe specific reaction instances as well as abstractions of reaction types. Three levels of abstraction are employed. The lowest level describes the partial order of atom types, which gives an explicit hierarchy at the atom level by specifying the degree of similarity between atoms. Following the atom level description is the structural level description. This uses a list of functional groups as structural features by which to characterize individual molecules. The structural characterization is then used to specify which molecules correspond to atoms in the reaction centre. The highest level of abstraction specifies physiochemical properties, which describe the function of the corresponding structure. The hierarchy therefore enables a richer description of a chemical reaction than a purely structural one (as with SMILES).

#### InfoChem CLASSIFY

The approaches used by Saller and co-workers to represent reactions underlies [75] and has inspired many of the approaches used for rule-based synthesis planning [76, 77]. The first step is to identify and extract the reaction centre, defined as a set of atoms that have changed their number of implicit hydrogens, valency, number of π-electrons, atomic charges, or if at least one connecting bond belongs to the reaction centre. Bonds are defined as belonging to the reaction centre if they are made or broken. In order to identify such changing atoms and bonds, a mapping is used to identify equivalent atoms in the reactants and products.

Regardless of the representation used, a key problem in the representation of chemical reactions is the identification of the reaction centre. One approach to reaction centre detection and atom mapping is finding the maximum common substructure (MCS) between reactant and product molecules. The determination of the MCS is an NP-complete problem, meaning that the solution is non-deterministic in polynomial time. Several reviews discuss these approaches and have been referenced for the interested reader [78–80].

Having identified the reaction centre, atom hash codes are calculated for all atoms belonging to the reaction centre using a modified Morgan algorithm [53]. The hash codes include the following atom properties: atom type, valence state, total number of bonded hydrogen atoms (implicit and explicit), number of π-electrons, aromaticity, and formal charges as per the reaction centre definition. The hash codes generated for each atom in the reaction centre are summed for all reactants and one product of a reaction to provide a unique representation of the reaction centre.

**Fig. 7:**
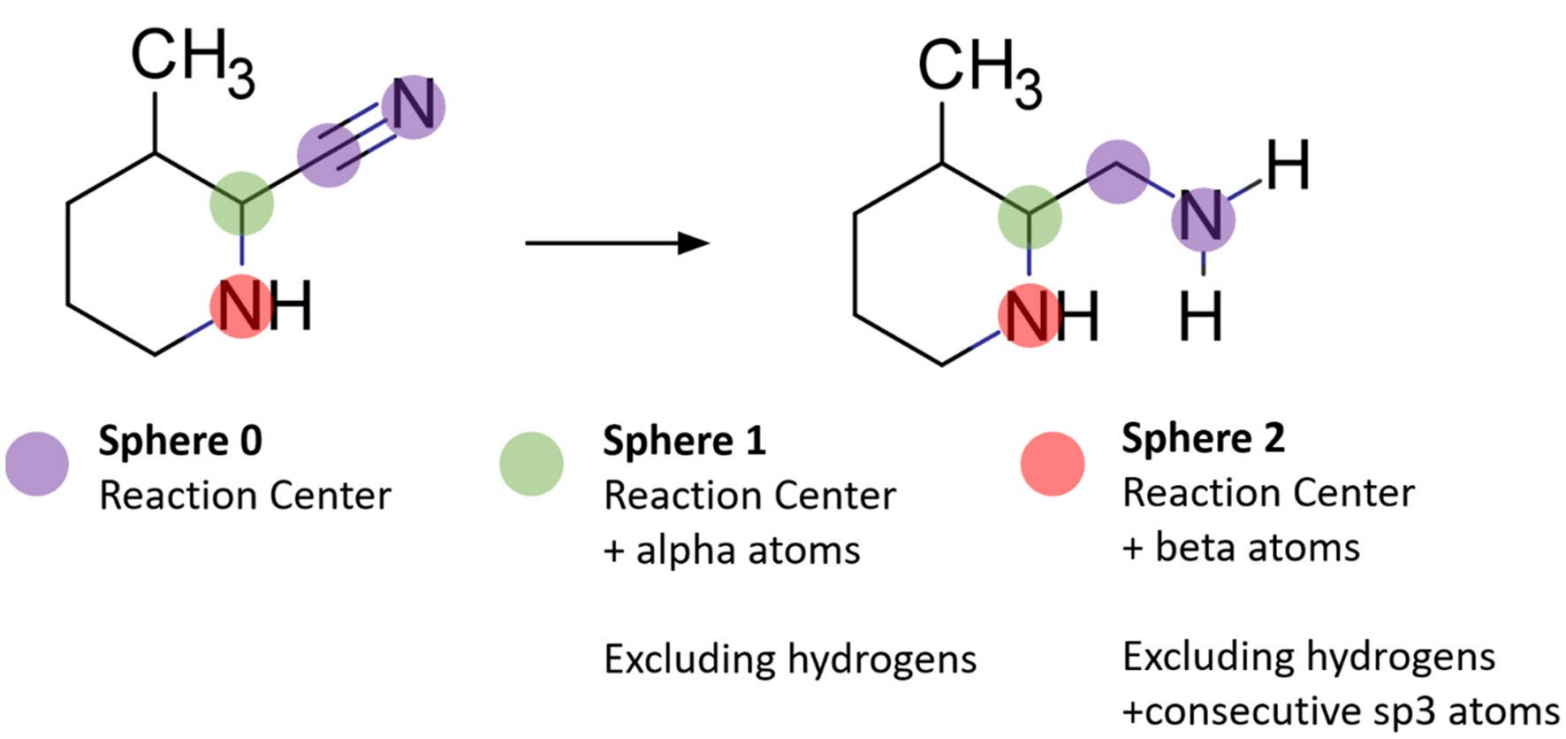
Atomic environments included in the description of the reaction centre. The reaction centre is used in calculations of atom hash codes for varying degrees of specificity

The description of the reaction centre can be extended to include the neighbouring chemical environment depending on the level of specificity required (Fig. 7). The reaction centre alone corresponds to a “broad” or more general description of the reaction, whereas inclusion of alpha atoms (atoms adjacent to those in the reaction centre) corresponds to a “medium” description of the reaction centre. Expanding the description to include the next set of adjacent atoms “narrows” the description of the reaction owing to increased specificity. The generated hash codes have been used in reaction classification and the approaches for reaction centre extraction utilized in a variety of synthetic planning tools.

#### Reaction fingerprints

Reaction fingerprints are vector representations of reactions. They specifically represent the structural changes taking place in the reaction centre. This information is captured by constructing fingerprints, such as the ECFP variant described previously, and taking the difference between the product and reactant vectors, optionally considering the agent. Schneider et al. [81] have used the difference fingerprint with the atom-pair variant to build a machine learning system for a 50-class reaction classification model. A similar approach to the computation of reaction vectors was described by Patel et al. [82] and has been used in de novo design and classification approaches [83]. The reaction fingerprint highlights an alternate approach to reaction centre detection and representation; however, it cannot be easily converted to a reaction graph. Lastly, the handling of stereochemistry has not been mentioned but is an active area of research [84].

## Representations for macromolecules

**Fig. 8:**
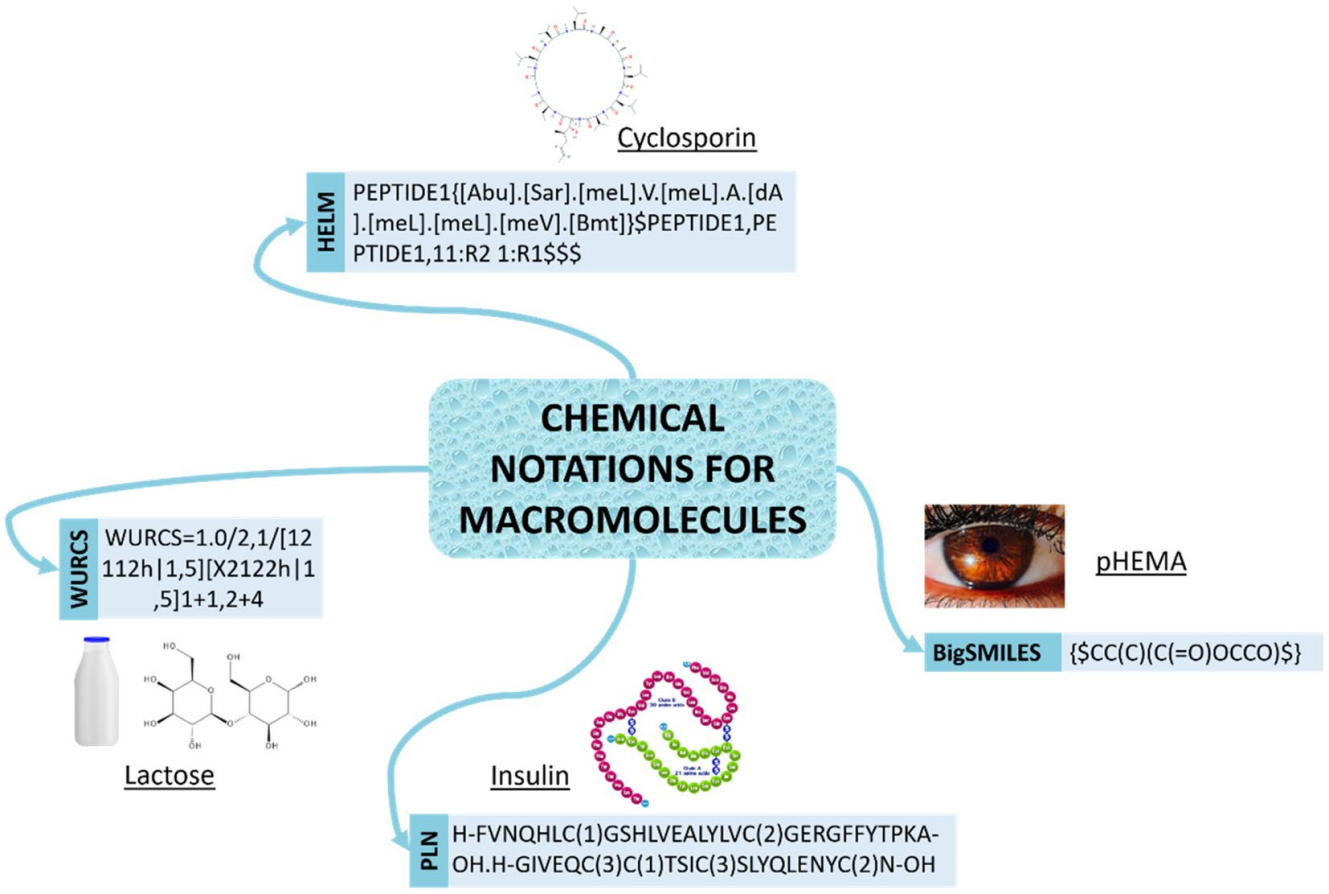
Example of linear notations for different types of macromolecules. Cyclosporin is an immunosuppressant medication and natural product. Lactose is a disaccharide used in the food industry. Insulin is a peptide hormone which regulates the metabolism of carbohydrates, fats, and protein. pHEMA or poly(2-hydroxyethyl methacrylate) is a polymer that forms hydrogel in water. Copolymers of pHEMA are used to make contact lenses

While there have been many advances in the representation of small molecules, in comparison very few studies [85] have addressed the representation of macromolecules, which are polymeric structures. In this section, we present representations made for biopolymers and bio-oligomers, like proteins and oligosaccharides, and synthetic polymers. The process of representing macromolecules can be hindered by the fact that, while many polymers are monodisperse (i.e. constituting monomers have the same chain length), others can be polydisperse, such that their stochastic nature results in an undefined chain length. Examples of macromolecules and their notations are shown on Fig. 8.

This section places itself at the interface between bioinformatics and cheminformatics. While in cheminformatics small molecules are described on the atomic-level, in bioinformatics, polymers, such as a proteins or polynucleotide molecules, are more commonly defined using their nucleotide and amino acid sequences. Representations which combine atomic and sequence information are presented here.

### Amino acid-based structures

The building blocks of peptides and proteins are amino acids (AAs), which are each made up of an amine group, a carboxyl group, and a side chain specific to each AA. AAs are commonly represented by a one-letter symbol, which commonly imply the L configuration for chiral AAs, or a three-letter abbreviation [86]. A limitation of the one-letter symbol is that while the Latin alphabet is large enough to describe the 20 AAs which appear in known genetic code, there are far more naturally occurring AAs.

#### Peptides

Peptides are chains of 2 to 50 AAs linked by peptide bonds. They can be antibiotics, immunosuppressants, or antitumor agents. This broad range of biological activity sparked the interest of the community.

A method named CHUCKLES [12] was developed in 1994 to infer SMILES of polymers from their sequences and vice versa. In cheminformatics, this method is particularly useful in inferring the SMILES from the peptide sequence, which is referred to as Forward Translation (FT). In FT, monomers sequences and SMILES are stored in a lookup table, with the SMILES excluding any atoms which would be involved in monomer bonding. For linear structures, SMILES corresponding to each residue are concatenated. In branched and cyclized structures, monomer indices are mapped to the SMILES, thus encoding for structures such as disulfide bridges. CHUCKLES is applicable to oligomeric structures and is used in BIOPEP-UWM [87]. An extension of CHUCKLES, CHORTLES, was designed to handle oligomeric mixtures. Two notations are well known for their ability to describe a broad range of macromolecules: the Hierarchical Editing Language for macromolecules (HELM) [14, 88] and the Self-Contained Sequence Representation (SCSR) [89]. Both representations were developed concurrently, the first one relying on SMILES and the second one on the v3000 Molfile format. SCSR was developed by BIOVIA which provides automated interconversion between HELM and SCSR.

**Fig. 9:**
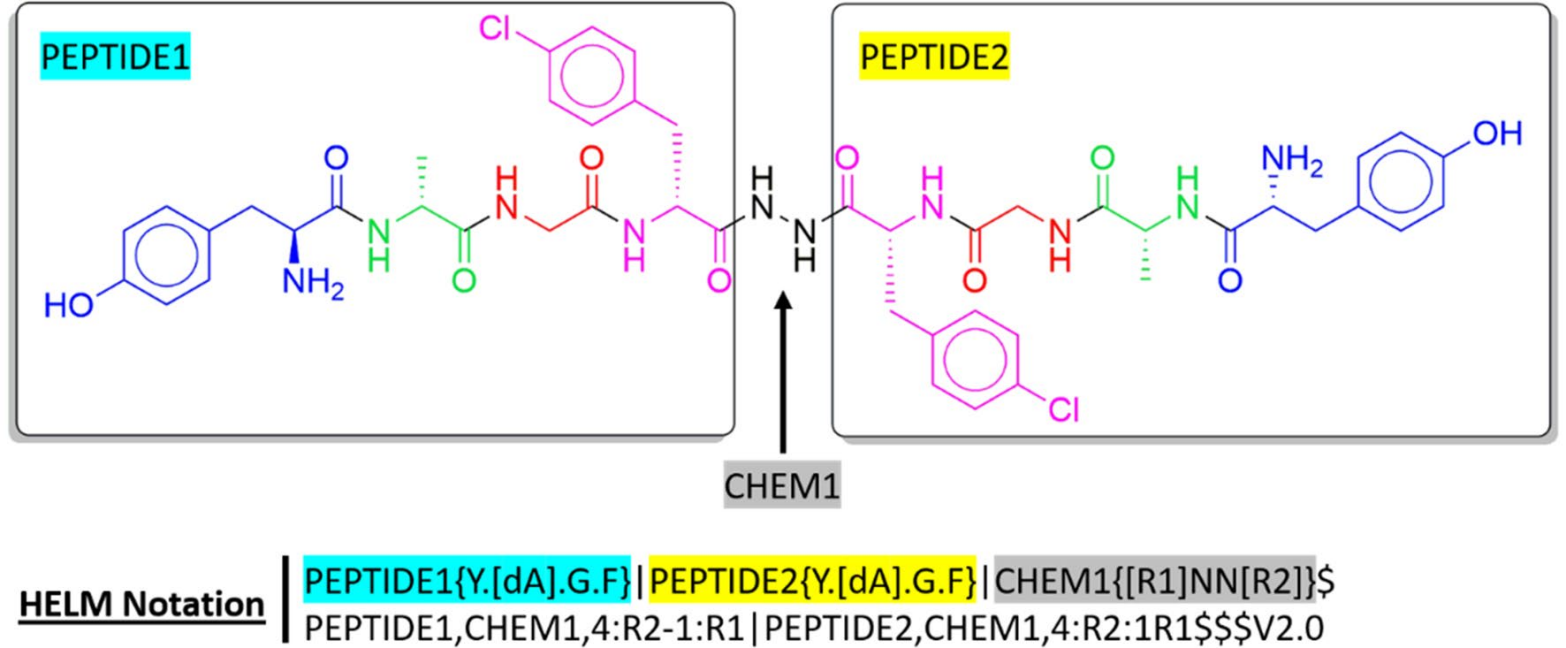
Graph and HELM representation of a biphalin analog. Amino acids are coloured coded as followed: blue, green, red, and pink for tyrosine (Y), alanine (A), glycine (G), and phenylalanine (F), respectively)

In the following lines, we provide further details on HELM, which was developed by Pfizer under the auspices of the Pistoia Alliance. The objective of the project was to design a system representing combinations of component structure types (e.g. peptides, antibodies, chemical modifiers). An example of HELM notation is shown in Fig. 9. Initially, HELM was limited to well-defined structures; however, HELM2 overcame this limitation and can describe polymer mixtures and free-form annotations. HELM represents monomers in a SMILES-like format, simple polymers using a simplified version of CHUCKLES and complex polymers using graphs. Its structure hierarchy follows the granularity of the structures: Complex Polymer, Simple Polymer, Monomer, and Atom. HELM is implemented in many pharmaceutical companies [90], in public databases (in 2016, ChEMBL21 contained 20,000 peptides annotated with HELM [91]), as well as in various packages and software such as RDKit (limited to peptides), ChemDraw, the Biomolecule Toolkit, ChemAxon, and Sugar&Splice, which can all encode for peptides, DNA, and RNA.

In comparison with purely atomic-based notations such as SMILES, biocheminformatics representations can facilitate the development of modified drug peptides. For example, the substitution of natural L-AA with D-AA can improve the oral bioavailability of a peptide [92]. Such modifications would be intuitive with HELM, which provides readability at the polymer level, whereas SMILES provides descriptions on the atomic level. While these methods constitute a step forward to a better understanding and unification of cheminformatics and bioinformatics, errors in the translation of peptide notation from biological into chemical language have been detected and practical solutions proposed [93].

#### Proteins

Proteins are polypeptides made up of 50 or more amino acids. They are generally biological targets; however, therapeutic protein drugs have been engineered [94]. The largest repository of 3D structures of proteins and nucleic acids is the Protein Data Bank (PDB) [17], which contains more than 150,000 structures. PDB entries contain the atomic coordinates of every atom in a protein structure as well as solvent molecules (if applicable). Each atom is identified by a sequential number, a specific atom name, the name and number of its corresponding residue, a one-letter code specifying the chain, its spatial coordinates (*x*, *y*, and *z*), and an occupancy and temperature factor. Furthermore, any notations mentioned in the *Peptide* section can be used for protein representations. In 2008, the Protein Line Notation (PLN) [16] was created by Biochemfusion and is implemented in PubChem. Pseudo-atoms were used to represent a simplified version of a residue structure, which enabled a lossless conversion between chemistry and sequence formats.

### Key macromolecules

Most drugs are small organic molecules. However, drugs can also be macromolecular in nature, such as glycans (also referred to as carbohydrates) [95] or synthetic polymers [96].

#### Glycans

Oligosaccharides and polysaccharides are glycans containing more than 3 and 20 monosaccharides (the smallest sugar unit), respectively. In drug discovery, glycans are interesting as receptors, small molecule glycomimetics, therapeutic glycopeptides, and vaccines. Glycan databases are used by carbohydrate researchers and structures are generally recorded using monosaccharide-based notations [97–100]. These representations do not allow for the analysis of the interactions between glycans and proteins using docking techniques, which require atom-based representations. Converter tools have been developed [101, 102] that translate these notations to atom-based representations. With the aim of creating a linear and unique notation for glycan data, compatible with the usage of the semantic web, the Web3 Unique Representation of Carbohydrate Structures (WURCS) [15] was developed and combined bioinformatics and cheminformatics features. The newest version of WURCS [103], used by the International Glycan Structure Repository GlyTouCan [104], encodes the following features: the main carbon backbone of a monosaccharide residue, the backbone modifications (i.e. atoms belonging to a monosaccharide which are not part of the backbone), and the linkage information between the backbone and a modification. The notation provides explicit anomeric information and can handle ambiguous monosaccharide structures (e.g. unknown ring closure or anomeric information). Currently, WURCS is implemented in many databases but unsupported by most cheminformatics software.

Independent representations have been developed to address specific challenges. Pillong and Schneider [105] published a representation of monosaccharides based on pharmacophoric properties. Bojar et al. [106] developed a language model based on natural language processing (NLP) providing information on glycans connectivity and composition.

#### Polymeric drugs

In the context of drug discovery, polymers are primarily used as drug deliverers. Nonetheless, some polymers have been used as active ingredients. Recently, the BigSMILES [107] syntax was introduced to encode for homopolymers, random- and block co-polymers, and molecules with different degrees of complexity in connectivity, such as linear polymers, ring polymers, and branched polymers. The stochastic unit of these polymers is identified by a pair of curly brackets. Repeated units are delimited by a comma and listed inside these brackets. Although BigSMILES are currently not canonical, a canonicalization scheme is under development. No application of this notation is available to this date; however, the development of polymeric drugs is expected to flourish [96], and ML models could be applied to aid in related studies.

## Graphical representations for molecules and macromolecules

The representations presented in the previous sections are designed for the storage and the cheminformatics analysis of compounds. In this section, representations which are made for direct visualization of compounds or/and their physicochemical properties are introduced.

### 2D depictions

**Fig. 10:**
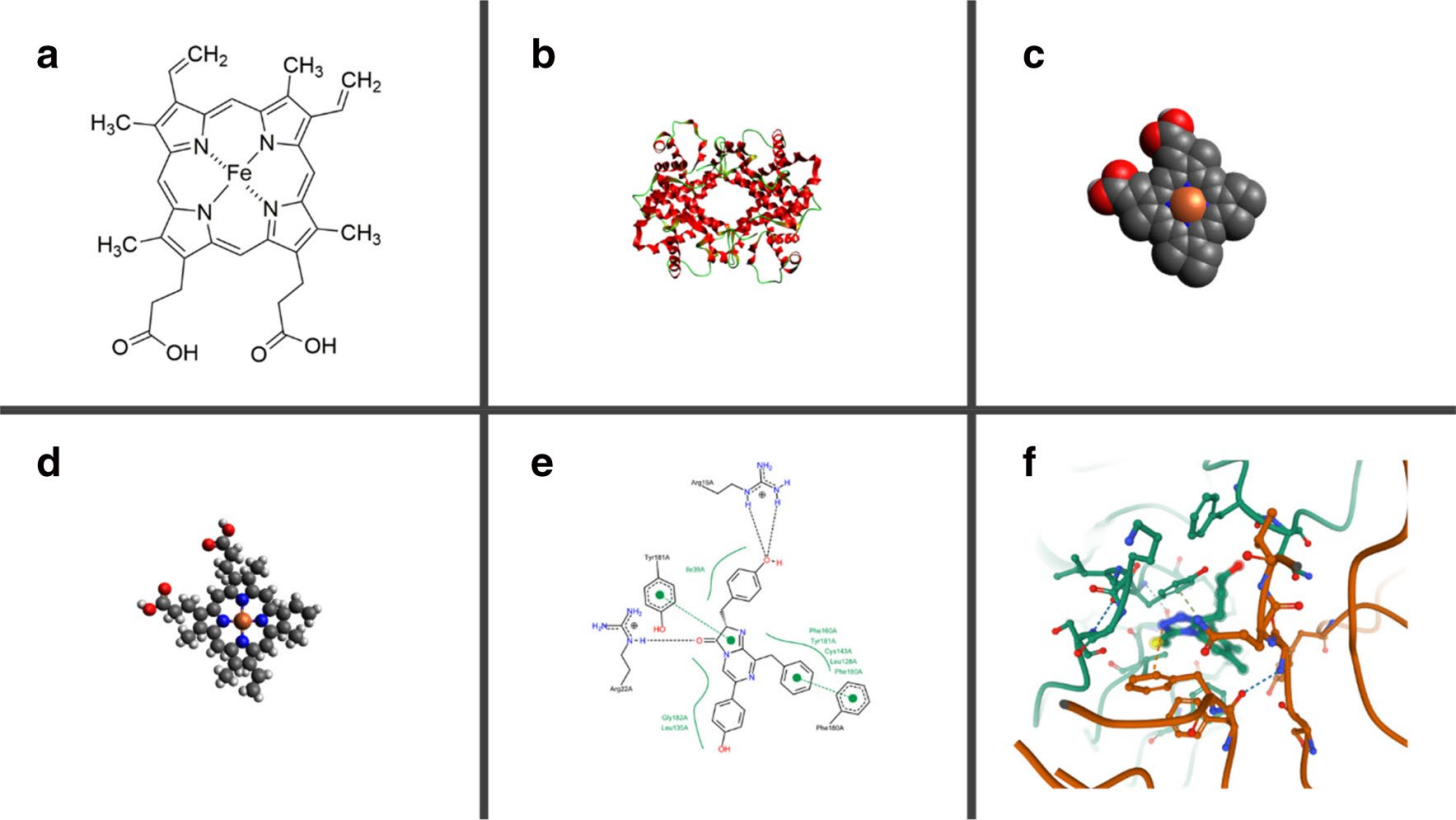
Examples of various molecules drawn using different display types. **b**–**d** Generated with Avogadro [32]. **a** Skeletal structure of the Fe-porphyrin subunit of haem B. **b** Ribbon diagram of haemoglobin. **c** Space-filling model of the Fe-porphyrin subunit of haem B. **d** Ball-and-stick model of the Fe-porphyrin subunit of haem B. Note the different orientations. **e** 2D visualization of protein–ligand interactions (PDB code: 2HPS). Reprinted with permission from [115]. Copyright 2020 American Chemical Society. **f** 3D visualization of protein–ligand interactions (PDB code: 6KYA)

Molecules as raster or vector images are very often represented by their skeletal structures, which are referred to as 2D depictions (Fig. 10a). Many difficulties can be encountered when generating 2D depictions related to the layout (e.g. orientation, overlap) and rendering (e.g. font, abbreviations, atom labels alignment) of the image. In 2008, the IUPAC issued recommendations for the standard display (typography, orientation of structure, etc.) of 2D depictions [108]. Such obstacles are overcome by a range of algorithms; however, as of now, none of them can perfectly display every chemical structure. This was exemplified in 2008 in a comparative study of 2 proprietary toolkits (Cactvs [109], used by PubChem, and Molinspiration [110]), and 3 open-source toolkits: RDKit, OASA [111], and CDK [112]. In 2017, improvements were done in CDK to depict stereochemistry more accurately and to solve atomic overlap [112]; on the latter point, the algorithm went from a heuristic approach to a refinement process. For more up-to-date details and examples of 2D depiction algorithms and their limitations, we refer the readers to a 2016 presentation by John Mayfield [113].

Apart from the 2D depictions of the structures themselves, molecules can be depicted in various ways for reactions and interaction studies (Fig. 10e). In the latter, the aim is to investigate the environment or the behaviour of a molecule rather than its structure. A specific 2D depiction worth mentioning is the Markush structure, especially useful in patents, which depicts a specific series of compounds. A Markush structure possesses a fixed core with one or several variable parts which can be described by -R groups, bonds, atoms, etc. For macromolecules, different types of depictions are needed as the visualization often focuses on the polymer or peptide structures rather than the atomic structure. Associated to HELM, the Pfizer Macromolecule Editor (PME) was developed to visualize polymer structures and calculate molecular properties. A notable nomenclature for the depiction of glycans is provided by the Consortium for Functional Glycomics [114].

### 3D depictions

Before the advent of computers, various molecular models were developed to visualize and manipulate molecules in 3D and were built by assembling balls and sticks made of materials such as plastics or metals. Nowadays, while physical molecular modelling kits are still used in educational environment to represent basic structures, visualization software has become a tool of choice for 3D graphical displays of molecules (examples of visualization software have been provided in the subsection *Introduction to the molecular graph representation*). The software Avogadro, PyMOL, and VMD all offer the popular representations *ball*-*and*-*stick*, *cartoon*, and *van der Waals (vdW),* as well as many independent representations. Each representation is useful for the visualization of specific properties, be it the structure coloured by atom types (Fig. 10d), the secondary biological structure (Fig. 10b), or the space-filling vdW spheres (Fig. 10c). The vdW spheres help visualize the surface on which the molecule can build interactions; using this depiction, interactions between proteins and ligands can be visualized in 3D (Fig. 10f). 3D depictions are especially useful in docking and mechanistic studies, while 2D depictions are standard in structure–activity investigations.

## AI applications within drug discovery using molecular representations

Most of the representations we have discussed above have seen widespread use within the fields of drug discovery and artificial intelligence. If there are any molecular representations which the reader feels were not discussed, it is because we, the authors, were not aware of the widespread use of that representation within our specialized fields of research. The omission of some representations may have been intentional, or it may have been due to the fast rate of change and developments in this field as the availability of useful datasets for drug discovery applications grows. Lastly, several concepts that were historically used may see a resurgence as they are adapted to suit current methods.

### Graph representations for small molecules

Despite this being a review of molecular representations, many of these representations are themselves used in *representation learning* applications within deep learning. Representation learning is the idea of learning an internal representation (e.g. a vector) for a given object (e.g. a molecule) and then using that internal representation for a predictive task. These internal representations are learned, meaning models can be trained to create them using classic techniques such as backpropagation in neural networks. With representation learning tasks, it is key to first identify a suitable input representation of a molecule that contains as much of the desired/necessary information to solve a problem as possible. Of the applications described below, any using deep neural network (DNN) architectures are essentially carrying out representation learning tasks, whereas classical ML methods such as random forests (RFs) and support vector machines do not operate by learning internal representations.

With the development of graph neural networks, a wave of recent work in drug discovery has focused on using the molecular graph representation directly for both property prediction and de novo design. As such, the molecular graph representation can be used for various applications within AI, and there is a large body of work discussing its use for molecular property prediction [26, 116–119], and, more recently, molecular graph generation [120–125] and synthesis prediction [126]. In most cases this is done through graph representation learning, by which a graph embedding is obtained from the full graph representation using a graph network [127, 128]; the learned graph embedding can be used as input to a property prediction model, such as a RF or DNN, in the same way a classic molecular fingerprint [66, 129, 130] is used. Until recently, more compact linear notations such as SMILES strings were favoured for many ML applications involving molecules, in part due to the larger memory requirement of molecular graph representations; this is, however, slowly changing. For two excellent reviews of deep learning applications in chemistry and drug discovery, we recommend [26] and [131]. For a good review on molecular generative models using AI, we recommend [132].

Another popular use of molecular graphs both within and outside drug discovery lies in part outside AI, where graphs are used as input for atomistic simulations (e.g. molecular dynamics) where atomic coordinates and periodic boundary conditions are used as the starting point for program-specific file formats which contain not only all atom coordinates, but also detailed bond information (e.g. bond length, dihedral angles, torsional angles) necessary to calculate the energy of a given molecular configuration using force fields. As such, the molecular graph representation has widespread use in molecular dynamics applications within drug discovery, such as docking, protein folding, and free energy perturbation calculations. These applications have been assisted by recent developments in AI [133, 134].

### Linear notations for small molecules

Popular applications of linear notations such as SMILES and molecular fingerprints are in molecular property prediction and QSAR. SMARTS patterns have been used to define substructures with the aim of selecting or eliminating associated compounds [135–137]. Additionally, the use of string representations such as SMILES has seen a lot of unexpected success in the field of de novo molecular design using tools from NLP. Data augmentation can be done for many applications using randomized SMILES [51, 138]. String representations have also seen success in property prediction using the learned latent space representations obtained using autoencoder frameworks [139]. As mentioned above, many of the aforementioned neural network models work by learning a vector representation for molecules in the training set, and using that learned representation to predict properties [116, 118, 140]; this is analogous to the older use of hashed fingerprint representations for molecular property prediction using traditional ML approaches, hence the term *learned fingerprints*.

### Representations for chemical reactions

Common applications of the reaction representations are in retrosynthesis and reaction prediction. This is an important field of research, as the synthesizability of proposed compounds is key to computational drug design and having suitable retrosynthesis tools would allow scientists to “close the loop” of AI-driven drug discovery. Many of these applications are also discussed in [26, 69].

### Representations for macromolecules

Popular applications of the macromolecular representations introduced in this work are in protein structure prediction, as having an accurate picture of a protein and the role it plays in a given disease can help scientists to develop molecules for the right target. Pillong and Schneider [141] successfully applied their pseudo-receptor model in a virtual screening study aiming to identify aminoglycoside scaffolds with antibacterial potential. The interactions between glycans and proteins have been investigated [142, 143] using ML. An important field of investigation linked to glycans is the prediction of glycosylation sites. Many tools were developed to infer such predictions and have been applied recently in the pipeline for the prediction of oncology drug targets [144] and the characterisation of the novel coronavirus (2019-nCoV) [145].

### Graphical representations for molecules and macromolecules

We previously showed how the process of visualizing molecules has become faster, more practical, and more enjoyable thanks to better computational tools. This process is still an important field of research for which virtual reality and 3D printing techniques have been developed. Moreover, as the need for harvesting the large amounts of published data grows, the demand for methods for easily mining structures from papers and patent data is also growing. Optical Character Recognition (OCR) systems, relying on a variety of ML and probabilistic pattern recognition techniques, were created to translate 2D depictions of chemical structures to standard chemical representations [146–148]. Nonetheless, the development of OCR systems can be hindered by the images’ resolutions, the computational interpretations of chemical abbreviations, and the nature of the image representation, which can be embedded in text, in figures containing multiple structures, or in reaction pathways, and can be represented as either a skeletal formula or a Markush structure.

### Discussion

At this point, it might become clear to the reader that many applications within drug discovery require multiple representations to be used simultaneously to solve a problem. For example, in protein structure prediction, one might start with the protein sequence, create a rudimentary 3D model of the structure, and then use advanced molecular dynamics methods to understand how the protein folds and what the final configuration/structure for the protein might be. The coordinates of the optimized protein structure (e.g. a PDB file) might then go on to be used in docking calculation, etc. Technical aspects may factor into the choice of representation(s) a researcher might make, such as the complexity of the method(s) for generating the representation(s), and if they are openly accessible.

It is interesting to note that some representations have held the test of time better than others. This can be partly explained by the evolution of computer technologies, which have improved in terms of storage capabilities, processor qualities, and parallel programming capabilities. Standard representations such as IUPAC-Dyson and WLN were sensible during their times and were made to be manipulated by humans, but difficult to work with on a computer. Computationally simpler representations are now frequently used. Furthermore, detailed representations which require greater computational time to compute (compared to molecular string representations) can nowadays be used; this is the case for hashed fingerprints. Another possible explanation for the endurance of certain molecular representation is that they are more human readable than others, and thus have been better received by the cheminformatics community. Lastly, another reason why some notations persist and others do not is that within different fields and subfields (e.g. cheminformatics, bioinformatics, or AI), different notations are often preferred for either historical or continuity reasons within groups.

## Conclusions

Molecules are complex structures and their representations must account not only for a wide variety of properties, such as stereochemistry and valence, but also for the different nature of these small molecules and macromolecules. The rise of cheminformatics and bioinformatics has led to a faster and more efficient drug discovery process as well as to a better understanding of molecular behaviour. In this review, we presented various popular notations and representations for small molecules, polymers, and proteins and their most common uses related to AI within computational drug discovery. We hope that this review will benefit practising cheminformaticians, students, and anyone else interested to learn more about the underlying molecular representations in cheminformatics that can be used in AI-driven drug discovery applications.

## Data Availability

Not applicable.

## Abbreviations

2D: 2-dimensional
3D: 3-dimensional
AA: Amino acids
AI: Artificial intelligence
CATS: Chemically Advanced Template Search
Ctab: Connection tables
CXSMILES: ChemAxon Extended SMILES
ECFPs: Extended Connectivity Fingerprints
FT: Forward Translation
HELM: Hierarchical Editing Language for Macromolecules
InChI: International Chemistry Identifier
IUPAC: International Union of Pure and Applied Chemistry
MDL: Molecular Design Limited, Inc
ML: Machine-learning
NLP: Natural Language Processing
OCR: Optical character recognition
PLN: Protein Line Notation
QSAR: Quantitative structure activity relationship
RDfiles: Reaction-Data Files
RInChI: Reaction InChI
RXN: Reaction
SMARTS: SMILES Arbitrary Target Specification
SMILES: Simplified Molecular Input Line Entry System
SMIRKS: SMILES ReaKtion Specification
WLN: Wiswesser Line Notation
WURCS: Web Unique Representation of Carbohydrate Structures

## Footnotes

1 Note that throughout this section, using the typical convention, bold italicized symbols are used to represent matrices and vectors, where an uppercase symbol specifically denotes a matrix (e.g. ***X***) and a lowercase symbol specifically denotes a vector (e.g. ***x***_***i***_). Furthermore, uppercase symbols that are italicized but not bolded are used to represent sets (e.g. *V*), whereas lowercase symbols that are italicized but not bolded are used to represent either a) items from a set (e.g. *v*_*i*_) or b) elements of a matrix (e.g. *a*_*ij*_), depending on context.

2 One-hot encoding is a widely used technique in AI to convert categorical data into numerical data using binary vectors, where a 1 indicates the presence of a quality and a 0 indicates the absence. See Fig. 1c; the rows of the node features matrix are examples of one-hot encodings.

3 List of scientists who proposed seven notation to IUPAC: M. Gordon, C. E. Kendall and W. H. T. Davson, W. Gruber, J. A. Silk, E. S. Cockburn, G. M. Dyson, G. K. Zipf and W. J. Wiswesser.
